# Necrological

**Published:** 1880-08

**Authors:** 


### NECROLOGICAL.

> "Death is the crown of life:
> Were death denied, poor man would live in vain."

Died on June 14th ultimo, at his residence, Pelham, Westchester county, N. Y., Dr. Richard Lewis Morris, in the 75th year of his age.

Died at Colorado Springs, Col., July 21, 1880, Boswell Preston, infant son of Dr. and Mrs. B. P. Anderson, of that city, aged eight months.

Only a few brief months ago we were pleased to announce the birth of this angel child, and congratulate the happy parents upon his advent to their hearts and home; but alas! the painful task of recording his early departure has arrived.

The insatiate archer chose him, a bright and shining mark, the dart was sped, and now the peace that reigned in a loving home, lies slain with his cherished form, in the all-consuming grave. But, thank God, there is another and brighter world than this!

"A world of pure delight, where he a gentle seraph bright,
Leans on the bosom of his God, the purchase of Redeeming blood."

Not lost, but only gone before.

